# Whooping cough dynamics in Chile (1932–2010): disease temporal fluctuations across a north-south gradient

**DOI:** 10.1186/s12879-015-1292-2

**Published:** 2015-12-30

**Authors:** Mauricio Lima, Sergio A. Estay, Rodrigo Fuentes, Paola Rubilar, Hélène Broutin, Gerardo Chowell-Puente

**Affiliations:** Departamento de Ecología, Pontificia Universidad Católica de Chile, Casilla 114-D, Santiago, 6513677 Chile; Center of Applied Ecology and Sustainability (CAPES), Santiago, Chile; Inst. de Ecología y Evolución, Facultad de Ciencias. Univ. Austral de Chile, Casilla 567, Valdivia, Chile; Departamento de Epidemiologia, Ministerio de Salud, Santiago, Chile; MIVEGEC, UMR CNRS 5290-IRD 224-UM1-UM2, 911, Avenue Agropolis BP 64501, 34394 Montpellier Cédex 5, France; School Public Health, Georgia State University, Atlanta, GA USA

**Keywords:** Pertussis, Latitudinal gradient, Susceptible recruitment, Cycles, Vaccination

## Abstract

**Background:**

The spatial-temporal dynamics of *Bordetella pertussis* remains as a highly interesting case in infectious disease epidemiology. Despite large-scale vaccination programs in place for over 50 years around the world, frequent outbreaks are still reported in many countries.

**Methods:**

Here, we use annual time series of pertussis incidence from the thirteen different regions of Chile (1952–2010) to study the spatial-temporal dynamics of Pertussis. The period 1975–1995 was characterized by a strong 4 year cycle, while the last two decades of the study period (1990–2010) were characterized by disease resurgence without significant periodic patterns.

**Results:**

During the first decades, differences in periodic patterns across regions can be explained by the differences in susceptible recruitment. The observed shift in periodicity from the period 1952–1974 to the period 1975–1995 across regions was relatively well predicted by the susceptible recruitment and population size. However, data on vaccination rates was not taken into account in this study.

**Conclusions:**

Our findings highlight how demography and population size have interacted with the immunization program in shaping periodicity along a unique latitudinal gradient. Widespread *B. pertussis* vaccination appears to lead to longer periodic dynamics, which is line with a reduction in *B. pertussis* transmission, but our findings indicate that regions characterized by both low birth rate and population size decreased in periodicity following immunization efforts.

**Electronic supplementary material:**

The online version of this article (doi:10.1186/s12879-015-1292-2) contains supplementary material, which is available to authorized users.

## Background

Whooping cough, mainly due to *Bordetella pertussis*, remains an important public health problem worldwide although large-scale vaccination campaigns have been carried out for over 50 years. For this purpose, it is crucial to increase our understanding of the interplay of changing susceptibility and demographic factors [1, 2]. Despite long-term vaccination efforts around the world, vaccination rates varied within and between countries, hence, pertussis remains endemic, and frequent outbreaks are still reported in many countries [3]. For instance, the United States experienced in 2012 one of the largest outbreaks of reported pertussis in 50 years [4]. Moreover, pertussis has been resurging in several countries with a long history of high levels of vaccination [5–7]. In particular, this increase has been associated with a shift in the average age of cases toward older age groups [7–10].

From a dynamical systems point of view, pertussis is characterized by a dominant cyclic pattern with a period ranging from ~3.5 to 5 years [11] during the vaccination period [1, 11]. This pattern is largely driven by variability in population immunity resulting from natural infection or vaccination campaigns as well as other factors including the rate of recruitment of new susceptible individuals in the population and the social contact network structure [2, 10, 11]. Importantly, the cyclic signature of pertussis dynamics has been reported in countries with different climatological conditions, socio-demographic characteristics and vaccination history [10]. This suggests that the determinants of pertussis dynamics such as the interplay of vaccination and demographic factors remains poorly understood. In this vein, most of the comparative analyses of pertussis dynamics come from time series data of particular countries [3, 11]. Furthermore, incidence data are typically analyzed at the national scale despite large geographic differences in socio-demographic conditions and spatial distribution of the population within the country’s borders [8, 12]. For example, the spatial dynamics of pertussis in Senegal are characterized by a transmission wave from urban centers toward rural areas, which suggests the importance of population size and density in epidemic timing [13]. This pattern is reminiscent of the traveling waves that exemplify measles in England and Wales [14, 15].

In this paper, we use long-term epidemiological time series data of pertussis incidence covering thirteen geographic regions of Chile together with statistical methods in order to analyze and disentangle the population dynamic patterns in Chile, a unique country spanning an extensive latitudinal gradient with a variety of climatic zones. Specifically, we explore the spatial and temporal dynamics of pertussis across this large latitudinal gradient and explore the influence of demographic factors, population size and vaccination on the observed dynamic patterns. The particular geographic features of Chile provide a unique opportunity to shed light on the transmission dynamics of pertussis in South America despite of the potential biases due to changes in notification rates and vaccination coverage along time and space.

## Methods

### Data

**Fig. 1:**
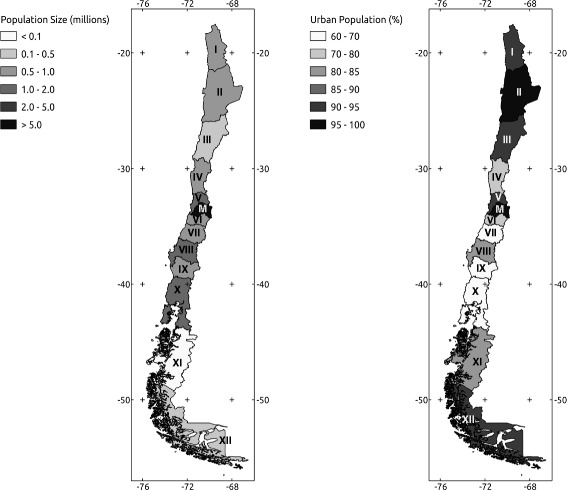
Map of Chile showing the distribution of the political regions and the demographic and urbanization characteristics of the country. Romans numbers are the different regional divisions (see Table 1). This map is made by one of the authors of the paper (Sergio A. Estay)

We obtained annual whooping cough mandatory notifications from the National Epidemiological Surveillance System (Department of Epidemiology, Ministry of Health, Chile) and from Epidemiological Statistics Yearbooks (Department of Statistics and Health Information, Ministry of Health, Chile). These data are freely available at the Ministry Web Site (http://www.deis.cl/). Definitions of cases are as followed; i) suspected case: a cough case having at least two weeks, with one of the following symptoms: paroxysms of coughing, inspiratory whoop, post tussive vomiting without other apparent cause, or cough of shorter duration if the features of the clinical case are depicted. In neonates and infants under 6 months, respiratory infection that causes apneas; ii) confirmed case: a suspected case that is laboratory confirmed or epidemiologically linked by the laboratory and iii) compatible or clinical case: suspected case which could not be demonstrated or confirmed by epidemiological association or confirmed at the laboratory. We obtained the annual time series of pertussis incidence at national scale for the period 1932–2010 and annual age-specific incidence at country level for the period 1952–2008. In addition, we obtained annual time series of pertussis incidence for each political region of Chile during the period 1952–2010 (Fig. 1). Demographic information were obtained from the Statistics National Institute of Chile (INE) including annual population size, birth rate (per 1.000) and infant mortality (deaths in infants per 1.000 live births) for each of the thirteen regions of the country (Fig. 1). Birth rates were corrected by infant mortality, for each political region of Chile. Vaccination in Chile started in 1955 using a whole-Cell DP (diphtheria/pertussis) vaccine during the period 1955–1974 and a whole cell DTP (diphtheria, tetanus and pertussis) vaccine during the period 1975–2006, and a whole cell pentavalent vaccine started to be used in 2007. The research reported in this manuscript was approved by the respective Committee of Bioethics and Biosecurity from the Faculty of Biological Sciences at Pontifical Catholic University of Chile (23/09/2013; CBB-196/2013).

### Change in variance

The algorithm of Inclán & Tiao [16] was used to detect changes in the temporal variance of pertussis incidence. This algorithm uses the cumulative sums of squares (D_k_) of a time series to identify retrospectively a change of variance. The central idea of this algorithm is that D_k_ (centred and normalized) has a Brownian bridge as an asymptotic null model. If the realized value of D_k_ exceeds the 95 % confidence interval for the expected maximum of the null model then this point is considered a true change of variance. However, to use this algorithm with ecological time series it is necessary to keep in mind some considerations. First, it is necessary to remove trends and autocorrelation from the time series. To remove trends, we replaced the original incidence data with the residual of the linear regression between the year and the incidence data plus the mean of the incidence series. To remove autocorrelation we used the residuals of an autoregressive model fitted to the detrended incidence data. Finally, because the ecological time are relatively short to use the asymptotic confidence intervals of the null model, the observed D_k_ values were multiplied by $\sqrt{T/2}$, where T is the length of the time series, for comparison with the asymptotic boundaries [16].

### Periodicity estimates

We used wavelet time series analysis for detecting the *pertussis* incidence periodicity [17]. This statistical methodology is well suited for extracting the relative weights of the different periodic components of a time series and their variation over time. Prior to conducting wavelet analyses, the time series data were log_e_ transformed and differenced in order to use the per capita rate of change of incidence as the response variable. We employed a continuous Morlet wavelet transform to the time series implemented in the *dplR* library in the R program (R Development Core Team 2008). In addition, we analyzed particular periods using a simple spectral density estimation implemented in the stat library in the R program (R Development Core Team 2008). More technical details on wavelet analysis are given in the Technical Appendix. The relationships between periodicity, change in periodicity, population size and the corrected birth rates were tested using linear regression models for the period 1952–1974 and 1975–1995.

## Results

### Changes in pertussis variability

Region-specific incidence time series showed reductions of variability (D_k_ values above confidence limits, Table 1). Maximum values of D_k_ statistics occurred in most regional time series (77 %) during the period 1974–1978 (Table 1). The only exceptions were region I (north region, latitude 19.73° S) and regions X and XI (41.49° and 46.42° S).

**Table 1:** Timing (years) in changes in the variance of the time series of Pertussis incidence across geographic regions of Chile

Region	Latitude (°S)	max D_k_	Year (max. Dk)
I	19.73	3,29	1960
II	23.54	2,76	1978
III	27.40	3,08	1974
IV	30.62	2,25	1977
V	32.78	2,66	1978
RM	33.60	2,51	1977
VI	34.44	2,48	1978
VII	35.62	2,66	1978
VIII	37.20	1,99	1978
IX	38.65	2,63	1977
X	41.49	2,36	1967
XI	46.42	2,63	1963
XII	52.46	2,85	1977

Latitude denotes the latitude of the centroid of each region, max D_k_ is the maximum value observed for the D_k_ statistic and Year (max D_k_) corresponding to the year when the maximum is observed

### Periodicity

**Fig. 2:**
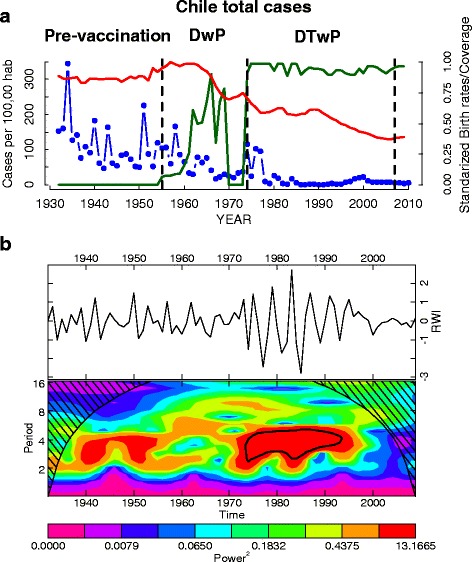
The annual temporal dynamics of pertussis at national level in Chile for the period 1932–2010. **a** The time series of incidence rate (cases/100000 hab.) for Chile (blue), the annual birth rates (normalized red line) and the annual vaccine coverage (green line); **b** The wavelet power spectrum of log_e_ pertussis incidence (differenced; top panel), the increasing spectrum intensity is from violet to red color; the dotted black curves show the statistically significant area (threshold 5 % confidence interval) the stripped area delimits the cone of influence (region not influenced by edge effects)

The time series at the national level is shown in Fig. 2a. The wavelet analysis of the aggregated annual time series showed clear changes in periodicity over time (Fig. 2b). During the pre-vaccination period (1932–1955) a 3–4 year cycle is observed. After the start of vaccination campaigns in 1955 no significant periodicity was detected until the 1970s, concomitant with an increase in birth rates (Fig. 2b). During the period 1975–1995, a 4-year cycle is detected and corresponding with a drop in birth rates and the start of mass vaccination campaigns in 1974 (Fig. 2a, b). From 1995, the incidence shows a positive trend (Fig. 2a, b).

The annual time series of pertussis incidence across geographic regions are presented in the left panel of Additional file 1: Figure S1. We found spatial and temporal variation in the periodicity of pertussis incidence across geographic regions of Chile (Additional file 1: Figure S1). In particular, the dominant dynamic signature was a strong 4-year cycle characterizing most of the incidence time series during the period 1975–1995 as indicated by wavelets analysis (Additional file 1: Figure S1). There were important differences across geographic regions. For example, regions (I, II, and III) localized at north in the Atacama desert showed some differences with the national pattern, the region II (the most urbanized and populated of the north) showed a clear 4-yr cycles since 1970 to nowadays. On the other hand, the region III (the least populated from the north) appears to cycle before the 70s and the cycles ended before the 90s (Additional file 1: Figure S1). These three northern regions did not show the positive trend in incidence observed at national level. Regions from central Chile (IV–VIII) showed the same pattern as the national time series, a strong 4-year periodic pattern since the start of mass vaccinations in 1974 until the middle 1990s (Additional file 1: Figure S1). In addition, all these regions showed a positive increasing trend in incidence during the last two decades and a sudden increase during 2011, which was particularly strong in regions VII and VIII (Additional file 1: Figure S1). Regions from southern Chile (IX, X, XI and XII) showed a different pattern of periodicity compared with the country (Additional file 1: Figure S1). There was a strong trend to show cyclic dynamics during the first two decades of the time series (1952–1975). In contrast with the general pattern, the southernmost two regions (XI and XII) did not show cyclic dynamics after the onset of mass vaccination in 1974. In particular, the region IX showed a different pattern characterized by a strong periodicity and a continuous negative trend of incidence (Additional file 1: Figure S1). During the period 1974–1995, the great majority of regions (10/13) showed a strong 4-year cycle (Additional file 1: Figure S1; Table 2) while nine regions showed a clear resurgent pattern during the last 20 years of our study period (Additional file 1: Figure S1; Table 2).

**Table 2:** Regional estimates of periodicity, susceptible recruitment rates (SR), population size, change in periodicity (∆ Periodicity), change in susceptible recruitment rates (∆ SR) and evidence for resurgence during the last 20 years

Region	Periodicity (1952–1974)	SR (1952–1974)	Population size (1952–1974)	SR (1975–1995)	Population size (1975–1995)	Periodicity (1975–1995)	∆ SR	∆ Periodicity	Resurgence
I	2	25.96	163517.8	19.23	411448.6	4.00	6.7	2	N
II	4	25.59	271331.9	19.80	502683.1	4.00	5.8	0	N
III	2	27.76	162502.6	19.20	258547.6	2.50	8.6	0.5	N
IV	2.7	26.23	370382.1	17.75	623274.8	4.00	8.5	1.3	Y
V	2.5	23.89	923194.1	15.85	1570163.6	4.00	8.0	1.5	Y
RM	2	25.83	3205838.7	17.10	6146877.5	4.00	8.7	2	Y
VI	2	25.78	494354.9	17.08	803304.7	4.00	8.7	2	Y
VII	3.3	25.86	659515.4	16.34	928979.4	4.00	9.5	0.7	Y
VIII	2.5	26.96	1309030.7	16.28	1900525.4	4.00	10.7	1.5	Y
IX	5	20.86	640086.6	16.15	885620.9	4.00	4.7	-1	N
X	4.5	23.08	787471.2	17.23	1064571.9	4.00	5.9	-0.5	Y
XI	3.5	25.03	50443.3	17.64	93718.0	1.00	7.4	-2.5	Y
XII	4	21.46	89211.4	15.08	151265.6	1.00	6.4	-3	Y

During the first two decades of the study period, the observed differences in periodicity between regions were significantly associated with differences in corrected birth rates (*R*^*2*^ = 0.53; *p* =0.0029). On the other hand, the observed changes in periodicity between the periods (1952–1974) and (1975–1995) across the country were relatively well predicted by population size (*R*^*2*^ = 0.51; *p* =0.0036). Population size showed positive effects on the change in periodicity between periods. Regions characterized by relatively high population sizes exhibited the higher increases in epidemic periodicity while those regions characterized by low population size displayed decreased periodicity after the onset of immunization efforts (Table 2).

### Age-specific incidence

**Fig. 3:**
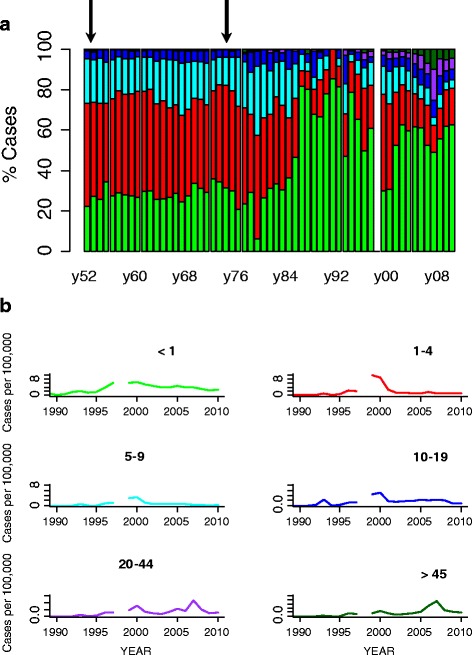
**a** Case percentage among age groups; infants (<1 year; green); preschool children (aged 1 – 4; red); primary school children (aged 5 – 9; cyan); adolescents (ages 10–19; blue), young adults (aged 20–44; magenta); older adults (>45; dark green). The onsets of the different vaccination programs are marked with the arrows. **b** Age specific pertussis incidence in Chile during the period of resurgence (1990–2010)

Age-specific changes in the composition of pertussis incidence in Chile during years 1952–2008 are depicted in Fig. 3. During the first vaccination period (DP cellular vaccine; 1955–1974) there was little variation in the distribution of cases across age groups despite large annual variation in prevalence and vaccination coverage (Fig. 3a). During this period, 28.7 % of the cases occurred among infants, 45 % among children 1–4 years, and 20.3 % among children 5–9 years, which is the typical pattern of age-specific incidence of pertussis (Fig. 3a). The onset of immunization with the DTP vaccine took place in 1975 and continued until 2006. In the mid-1980s a large change in the age-specific incidence occurred (Fig. 3b). Specifically, there was a change in the age-specific incidence pattern toward children < 1 year and young and older adults (Fig. 3a, b, Table 3). During the last two decades incidence rates increased across age groups in particular among infants (<1 year), adults aged 20–44 years, and adults >45 years (Table 3).

**Table 3:** Age-specific incidence rates of pertussis for different time periods at the national level in Chile

Years	I (<1 y)	I (1–4 y)	I (5–9 y)	I (10–19 y)	I (20–44 y)	I (>45 y)
1952–1960	24.80	42.90	17.43	3.92	0.32	0.14
1961–1970	11.35	18.82	8.56	1.95	0.18	0.03
1971–1980	12.78	22.45	9.02	1.94	0.19	0.17
1981–1990	1.84	1.53	0.98	0.40	0.04	0.01
1991–2000	3.25	2.78	1.01	0.42	0.21	0.07
2001–2010	3.44	1.89	0.73	0.41	0.30	0.16

Numbers in parenthesis are the age-classes in years

## Discussion

We have analyzed the dynamics and periodicity patterns of pertussis incidence in Chile by making use of almost 60 years of data along a unique latitudinal gradient in Chile. In fact, to the best of our knowledge, this is the first large-scale comparative analysis based on a latitudinal gradient of pertussis incidence. The same comparative approach has been applied to understand the global patterns of pertussis dynamics across different countries [3, 11]. One interesting component of our analysis is the relatively homogeneity of the epidemiological data originating from a consistent source of information and methodology. However, we are aware that despite having a single source of information (Chilean Ministry of Health), the great temporal and spatial extent of the data are sources of error and bias in aspects of notification, diagnostic tools and vaccination coverage.

Our findings underscore transient dynamic changes observed in time and space across an extensive latitudinal gradient. Specifically, three different dynamic behaviors can be distinguished at the national and regional scales namely high frequency dynamics, 4–5 year cycles and a resurgent pattern occurring during the last 20 years. During the pre-vaccination period (1932–1955) pertussis dynamics showed a weak cyclic behavior with a 2–3 years period, as predicted by theory the increase in birth rates observed among 1955–1965 produced a decrease in the inter-epidemic periods [18, 19] due to the increase of the susceptible pool. This dynamic change occurred despite the onset of vaccination in 1955. However, the first vaccination period in Chile (1955–1974) was characterized by a low coverage rate of two doses of a DP whole cell vaccine [20]. Not surprisingly, during this period the reduction in mean incidence rates was weak and pertussis dynamics seems to be driven by birth rates. The most important change in the variance occurred at the beginning of the 1970s, which is the result of a dramatic and synchronized reduction in the mean incidence across the country. During this period there were changes in the type of pertussis vaccine, the corresponding coverage levels and demographic characteristics in Chile. For example, in 1974, the vaccination program was initiated with three doses of the DTwP type vaccine and a vaccination program at national level that increased coverage [20]. A previous study based on eight countries with long epidemiological records showed that vaccination increased the periodicity of pertussis outbreaks [3, 11], which supports the idea that immunization plays an important role in decreasing transmission [21]. Nevertheless, Chile experienced important reductions in birth rates during this period; birth rates peaked in 1960 and declined by almost 40 % during the first half of the 1970s. Therefore, it is difficult to assess the effect of the relative contribution of immunization and demographic factors on the dramatic change in mean incidence rates and triggering 4-year periodic cycles.

The differences in pertussis dynamics observed along the regions appear to reflect the large climatic, social and demographic differences of the country and potentially also the vaccination coverage differences (data not available). For example, central Chile (regions IV–VIII) comprises about 70 % of the total population including the largest cities (Santiago, Viña del Mar, Valparaiso and Concepcion). These regions exhibited very similar dynamics; the strong 4-years cycles observed during the period 1974–1995 are associated with an important reduction in transmission rates caused by the increase in vaccination coverage, the addition of a third dose [20] and the decrease in birth rates. On the other hand, the extreme northern and southern regions characterized by lower population sizes and lower birth rates showed cyclic dynamics. The theory of infectious diseases predicts that outbreak periodicity is mainly driven by birth rates and vaccine coverage [18, 19, 22]. Consequently, outbreak periodicity appears to be driven by the susceptible recruitment dynamics (birth rates and vaccination coverage). Nevertheless, other studies have emphasized the role of waning immunity as the main driver of susceptible recruitment dynamics and outbreak periodicity in pertussis [23, 24]. In Chile, during the first period of vaccination (1955–1974) the periodicity of the outbreaks appears to be clearly influenced by demography (birth and infant mortality rates). In particular, the regions characterized by low birth rates exhibited longer periodic patterns, which is in line with theoretical results [18] and the observed patterns [3, 11]. However, the interaction between the different factors generating the dynamics of pertussis is still not clearly understood. For example, the immunization program in Chile increased the periodicity of the outbreaks in those regions characterized by high population size, while decreased the periodicity, especially in southern regions with low population sizes. This result is intriguing and poses some interesting questions about the complex dynamics of this infectious disease. The 3–to 4-year periodicity trend in pertussis dynamics has been observed in countries with very different vaccination levels and socio-demographic conditions [3], suggesting that other factors are operating beyond the role of susceptible recruitment and vaccination coverage [3], such as, population density, urbanization and the degree of biases in the notification of the disease. In fact, we found that in northern and southern Chile the periodicity of pertussis decreased following vaccination efforts. These particular regions were characterized by low population size and low birth rates, but because we have no information about vaccination rates among regions the causal factors are only speculative. Importantly, despite the existence of major demographic and social differences between regions, no effects were observed in the magnitude of the incidence rates associated with these factors.

During the last decade the resurgence of pertussis has been the subject of considerable attention, especially in countries with a long history of vaccination [5, 8, 25–29]. In general, the resurgence has been characterized by an increase in the mean age of infection [8, 11, 25–29]. Our analysis determined that the increase of pertussis incidence in Chile started around twenty years ago and displays differences with other countries. For example, in Canada, Sweden and USA, pertussis resurgence was driven by an increase in the number of preteens and teens [8, 25–27]. In Chile, the corresponding increase is in infants (<1 year old) and adults (20–44 and >45). The introduction of the DTPw vaccine in 1974 caused a dramatic reduction in incidence in the age-group 1–9, but the effects were less marked on the infants. This shift toward infants was also reported in Sweden [7], but the resurgent era in USA and Canada was characterized by about half of the cases reported in children > 10 year old [4, 8, 25, 26]. Different plausible hypotheses have been proposed for explaining this resurgent pattern and age distribution shift in highly immunized populations; evolution of *Bordetella pertussis* [30], improvements in the recognition of the disease in adolescents and adults [26, 27], waning of vaccine-induced immunity [8, 25–29], and the role of contact networks in highly immunized countries [7]. Moreover, the study by Rohani *et al*. [7] suggested that the social contact network can be the key piece for understanding the epidemiology of *B. pertussis*. The number of daily contacts among the different age classes could be an important factor for determining the epidemiology of *B. pertussis* [7, 31]. On the other hand, the increase of pertussis incidence was closely related with the regional geographic structure of Chile. Northern regions did not show any evidence of increases during the last 20 years, which is in contrast to the observed pattern in central and southern regions of Chile. In fact, the most important increases in pertussis were observed in the southern regions (VIII, X and XI).

## Conclusion

This comparative study of *B. pertussis* along a latitudinal gradient from Chile illustrates the regional dynamic patterns of this important disease. We related changes in pertussis periodicity and incidence trends along a latitudinal gradient with interactions between demography and population size. Widespread *B. pertussis* vaccination appears to lead to longer periodic dynamics, which is congruent with a reduction in *B. pertussis* transmission and herd immunity effect, but we detected that regions characterized by low birth rates and population size decreased in periodicity after immunization campaigns. This finding underscores the complex transmission dynamics of pertussis and the need for further studies to disentangle the links between transmission variables, susceptible recruitment and vaccination. In summary, we have characterized the geographical variation in pertussis dynamics along a latitudinal gradient with regional demographic variability. More studies based on high resolution spatial-temporal data including demographic, vaccination cover, good clinical and microbiological data covering extensive geographic areas are warranted to increase our understanding of the complex epidemiological dynamics of pertussis in Chile.

## Additional file

Additional file 1: The annual temporal dynamics of pertussis at regional level in Chile for the period 1952–2010. Left panel) The log transformed time series of incidence rate (cases/100000 hab.) for each region of Chile; Right panel) The wavelet power spectrum of loge pertussis incidence (differenced; top panel), the increasing spectrum intensity is from violet to red color; the dotted black curves show the statistically significant area (threshold 5 % confidence interval); the stripped area delimits the cone of influence (region not influenced by edge effects) (PPTX 1519 kb)
