# Supplementary material for: Whooping cough dynamics in Chile (1932–2010): disease temporal fluctuations across a north-south gradient
Source: BMC Infect Dis. 2015 Dec 30;15:590. doi: 10.1186/s12879-015-1292-2 (PMC4696083; doi:10.1186/s12879-015-1292-2)
Supplement: Additional file 1: — The annual temporal dynamics of pertussis at regional level in Chile for the period 1952–2010. Left panel) The log transformed time series of incidence rate (cases/100000 hab.) for each region of Chile; Right panel) The wavelet power spectrum of loge pertussis incidence (differenced; top panel), the increasing spectrum intensity is from violet to red color; the dotted black curves show the statistically significant area (threshold 5 % confidence interval); the stripped area delimits the cone of influence (region not influenced by edge effects) (PPTX 1519 kb) [file 12879_2015_1292_MOESM1_ESM.pptx]

## Slide 1
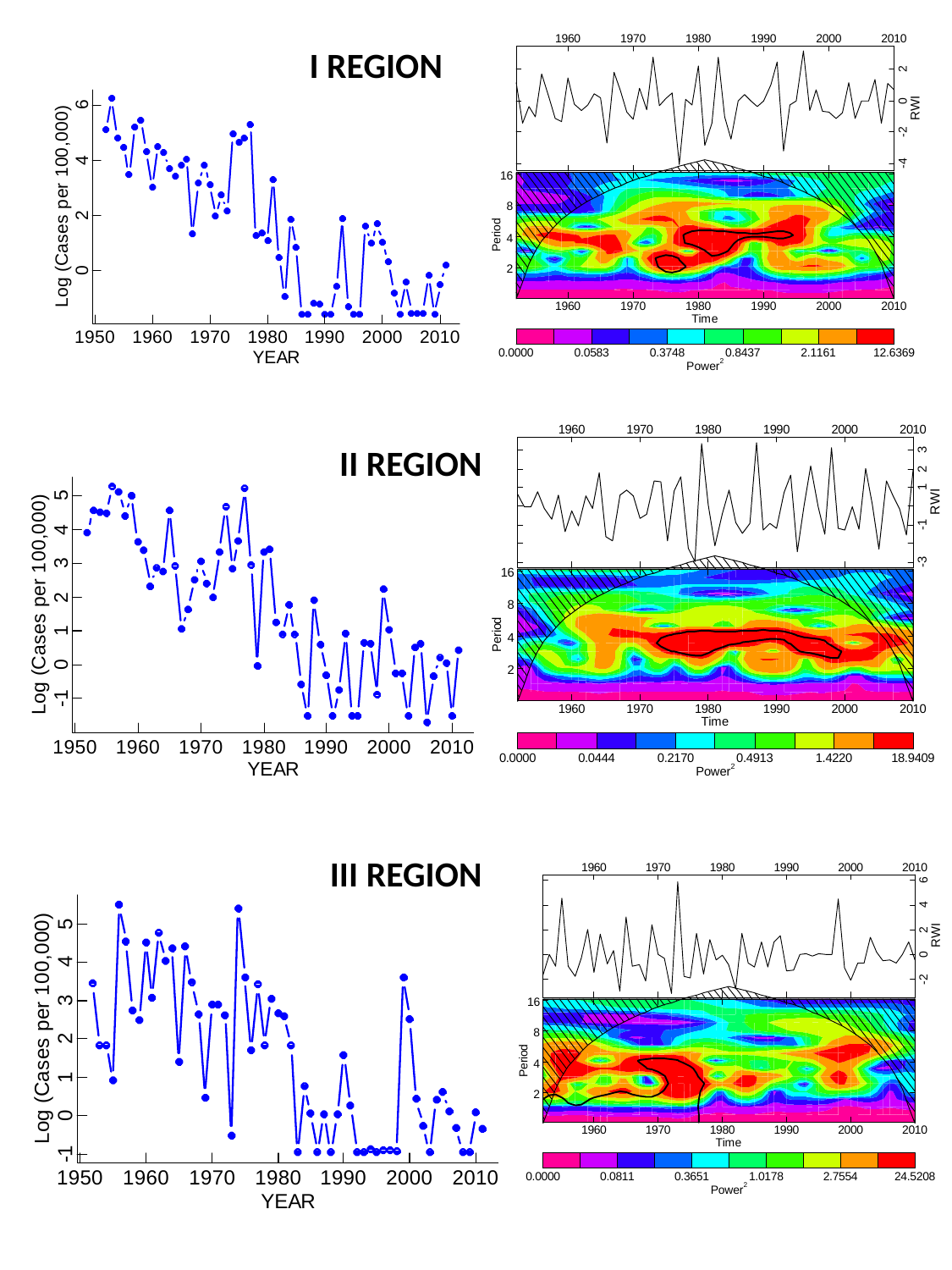

I REGION
II REGION
III REGION

## Slide 2
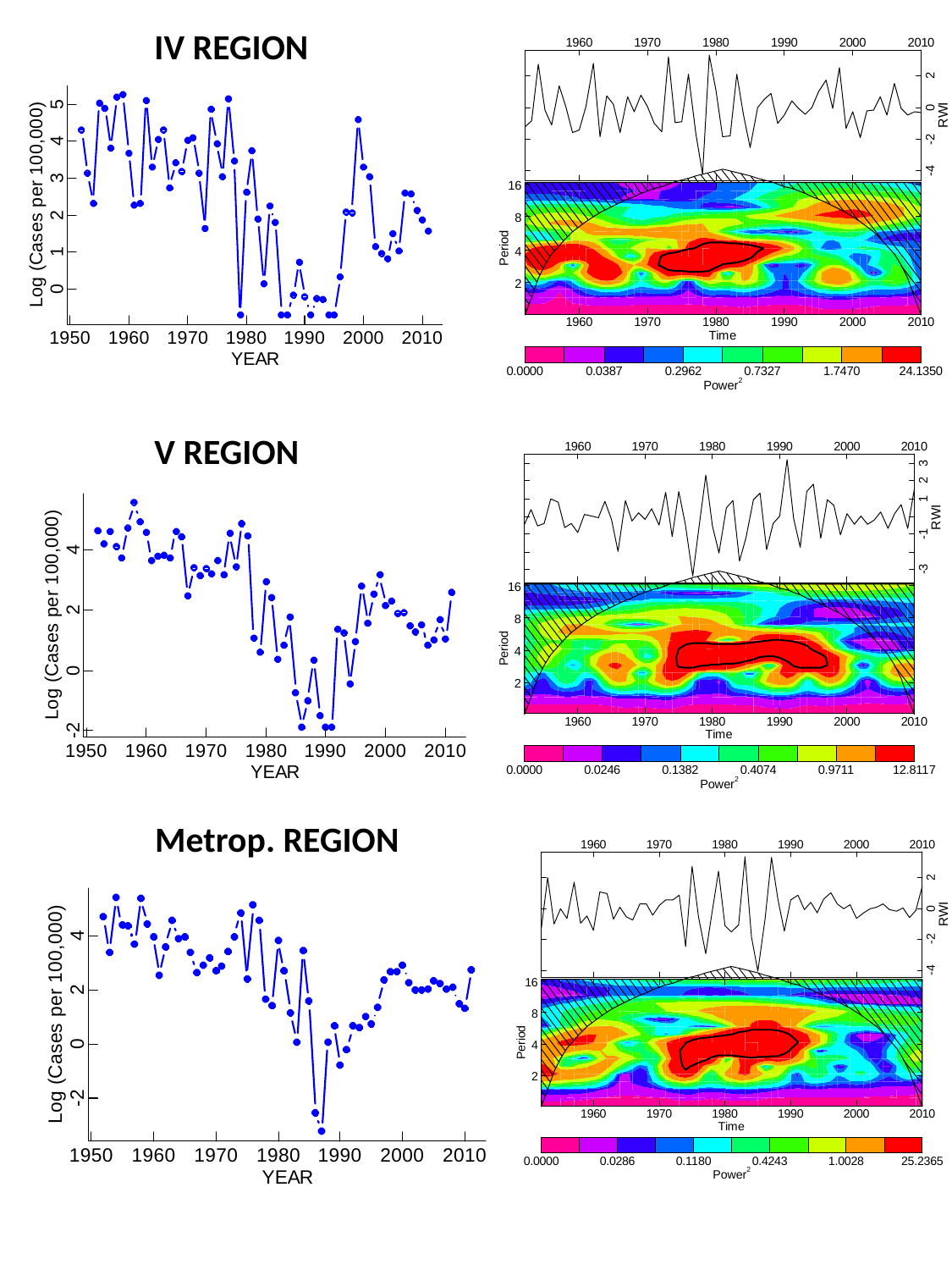

IV REGION
V REGION
Metrop. REGION

## Slide 3
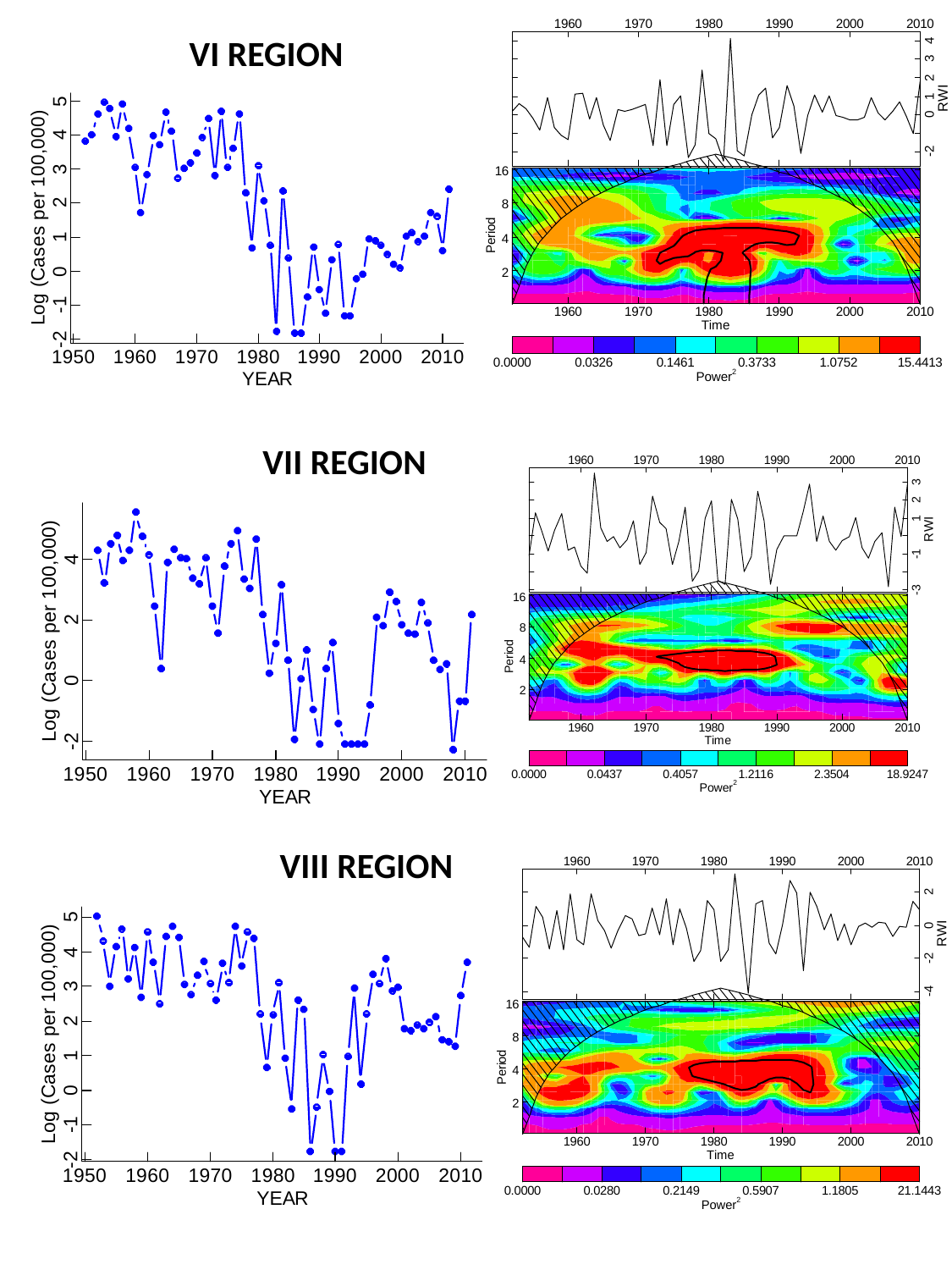

VI REGION
VII REGION
VIII REGION

## Slide 4
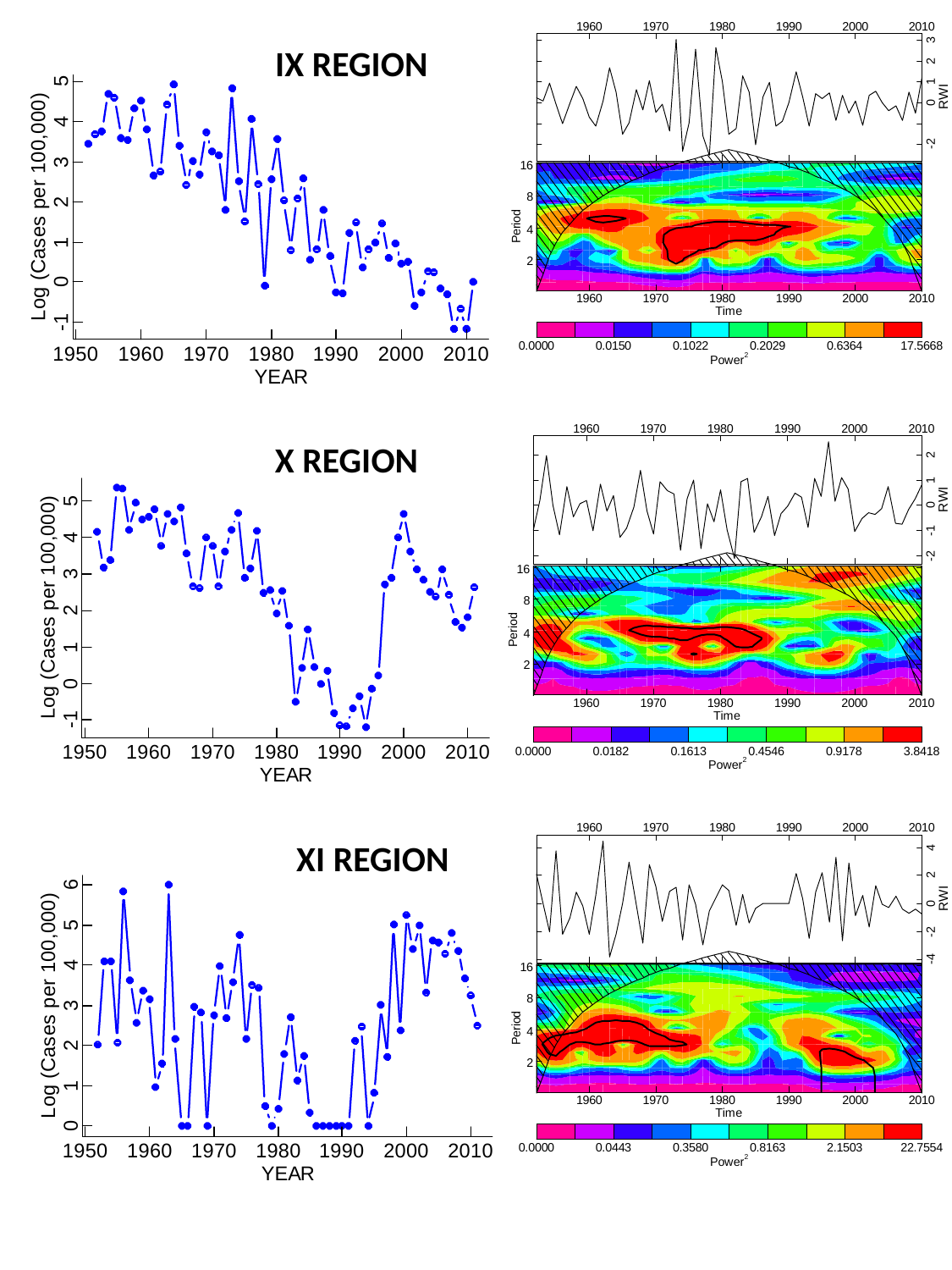

IX REGION
X REGION
XI REGION

## Slide 5
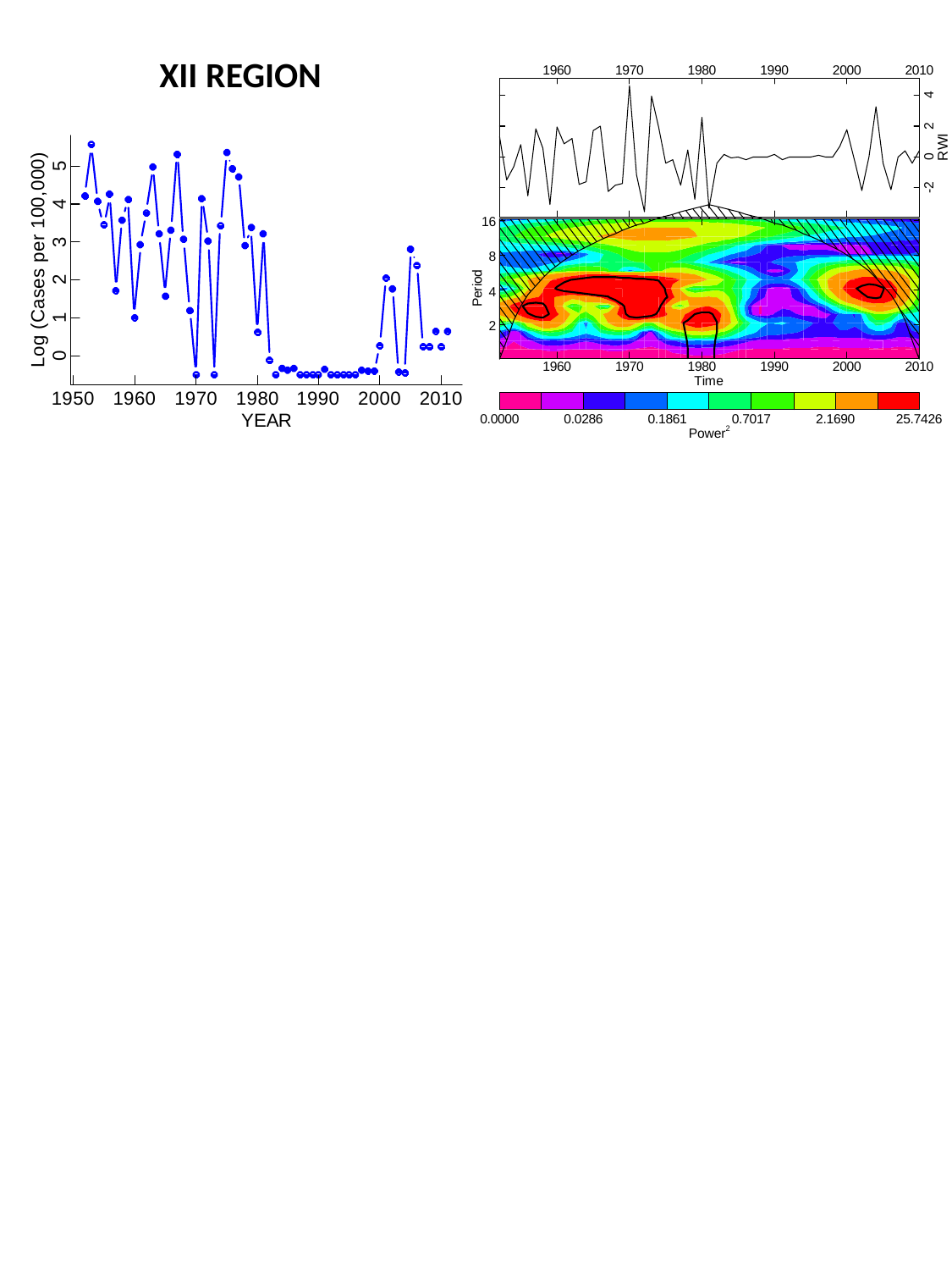

XII REGION
